# Antimicrobial Susceptibility and Detection of Virulence-Associated Genes in *Escherichia coli* Strains Isolated from Commercial Broilers

**DOI:** 10.3390/antibiotics10111303

**Published:** 2021-10-26

**Authors:** Tímea Kocúreková, Lívia Karahutová, Dobroslava Bujňáková

**Affiliations:** 1 Centre of Biosciences of the Slovak Academy of Sciences, Institute of Animal Physiology, Šoltésovej 4-6, 040 01 Košice, Slovakia or 16202@student.uvlf.sk (T.K.); karahutova@saske.sk (L.K.); 2 University of Veterinary Medicine and Pharmacy in Košice, Komenského 73, 040 01 Košice, Slovakia

**Keywords:** *E. coli*, broilers, iron uptake, phylogenetic groups, virulence-associated genes, antimicrobial resistance

## Abstract

The aim of this study was to investigate the presence of iron-uptake and virulence genes, antibiotic resistance profiles, and phylogenetic relatedness in 115 *Escherichia coli* (*E. coli*) strains isolated from broilers in Slovakia and to determine their potential threat to human health. The most frequent phylogroups were B1 (37%) and A (21%), and 33.9% strains were included in pathogenic groups. The commonly observed iron-uptake genes were *feoB* (94%), *sitA* (83%), and *iutA* (58%). Protectins (*iss, kpsMTII*) were identified in 30% of samples. Four percent of B2-associated broilers carried the *papC* (P fimbria) gene connected with upper urinary tract infection. The dominant resistance was to tetracycline (49%), ampicillin (66%), ampicillin + sulbactam (27%), ciprofloxacin (61%), and trimethoprim + sulfonamide (34%); moreover, sporadically occurring resistance to cephalosporins, aminoglycosides, fluoroquinolones, and polypeptide colistin was observed. Genotypic analysis of resistance revealed the presence of *bla_CTX-M-1_* and *bla_CTX-M-2_* in two isolates from broilers. Commercial broilers can be reservoirs of virulent and resistant genes as well as *E. coli* causing (extra-)intestinal infections, which can be a potential threat to humans via direct contact and food.

## 1. Introduction

The presence of potentially pathogenic *E. coli* strains in bird faeces may pose some risks to other animals and humans. In birds, *E. coli* isolates containing virulence factors are designated as avian pathogenic *E. coli* (APEC), which cause avian colibacillosis, and together with uropathogenic *E. coli* (UPEC) and *E. coli* causing meningitis in the newborn (NMEC), they belong among the extra-intestinal *E. coli* (ExPEC) strains. Since all ExPEC strains present the same phylogenetic origin and a notable degree of overlap in serogroups, sequence types (STs), and virulence-associated genes, several studies have suggested their zoonotic potential [1,2].

Another health concern is the presence of antibiotic-resistant bacteria, an emerging global problem in human and veterinary medicine and one of the main challenges for the twenty-first century. Bacteria can become resistant to antibiotics as a result of efficient horizontal gene-transfer mechanisms through mobile genetic elements such as plasmids, transposons, and integrons [3], the latter of which are able to integrate or excise gene cassettes in their structures.

Consequently, the presence of such pathogenic drug-resistant bacteria poses a public health threat. Humans can be infected due to non-compliance of hygiene procedures, such as washing hands with soap after manual manipulation with poultry and not wearing protective clothing and shoes on farms (it is possible to transfer faeces on shoes and contaminate the domestic environment). The other possibility is foodborne infections, because of contamination of meat during slaughtering or eggs during laying and subsequent insufficient heat treatment of these contaminated products [4].

Numerous studies on phylogenetic groups, virulence-associated genes, and antimicrobial resistance in APEC isolates from farm poultry derived from various colibacillosis have been conducted in many countries, to prevent drug-resistant pathogenic strains reaching consumer foodstuffs [2]. These pathogenic strains result generally from commensals by the acquisition of infectious capacity through horizontal transfer of virulence genes [5]. In order to better understand the risks of emergence of antibiotic-resistant pathogens from healthy animal reservoirs, it is necessary to know the presence and prevalence of virulence factors and antibiotic resistance in faecal commensal *E. coli*. In this context, the aim of our study was to analyse the distribution of phylogenetic groups and the occurrence of virulence factors and antimicrobial resistance in faecal *E. coli* strains isolated from healthy broilers kept on commercial farms in Slovakia.

## 2. Results

A total of 115 *E. coli* strains found in asymptomatic broilers from five commercial poultry farms were included in this study.

### 2.1. Clermont’s Phylogenetic Typing

Of the assumed commensal strains (*n* = 115) isolated from asymptomatic broilers, 21% belonged in phylogroup A; 37% in B1; 17% in D and F phylogroups and clade I (6 versus 4%); and 6% of isolates belonged in group B2. Eight isolates remained unknown.

### 2.2. Detection of Iron-Uptake and Virulence-Associated Genes (VGs)

A total of eight genes ascribed to the strains for iron uptake (*feoB, sitA, irp2, fyuA, iutA, iha, iroN,* and *ireA*) and six virulence-associated genes (*tsh, papC, cvaC, iss, kpsMTII,* and *ibeA*) were the subject of our study.

The most common iron-uptake genes observed in broilers were *feoB* (94%), *sitA* (83%), and *iutA* (58%). Other genes for iron transport were represented in lower percentages, namely, *irp2* = 22%, *fyuA* = 14%, *iha* = 4%, and *iroN* = 28%. The *ireA* gene was not detected in any sample.

Genes encoding protectins (*iss, kpsMTII*) were identified in an average of 30% of broiler samples (*iss*–34, *kpsMTII*–35 isolates). The presence of a gene encoding P fimbriae (*papC*), which is strongly connected with upper urinary tract infections, was confirmed in 4% of B2-associated *E. coli*.

Our isolates were sorted into the ExPEC subpathotype according to the presence of extra genes as described by Johnson et al. [6] (≥2 VGs: *papA/papC; sfa/foc; afa/dra; kpsMTII; iutA*). APEC diagnostic approaches (ExPEC + ≥4 VGs: *kpsMTII; iss; tsh; iutA/fyuA; sfa/foc/papA/papC/papEF*) were used as described by Stromberg et al. [7] and based on criteria according to Spurbeck et al. [8], whereby the strains were defined as UPEC (≥2 VGs: *chuA; fyuA; vat; yfcV*). Based on the established criteria for pathotype definition, twenty-five broiler isolates (21.7%) possessed ExPEC characteristics; eight (7.0%) were defined as UPEC and seven (6.1%) as APEC. Table 1 moreover shows the distribution of individual pathotypes related to phylogenetic groups, which implies that UPEC is associated only with B2 and D phylogroups.

### 2.3. Antimicrobial Resistance

**Figure 1:**
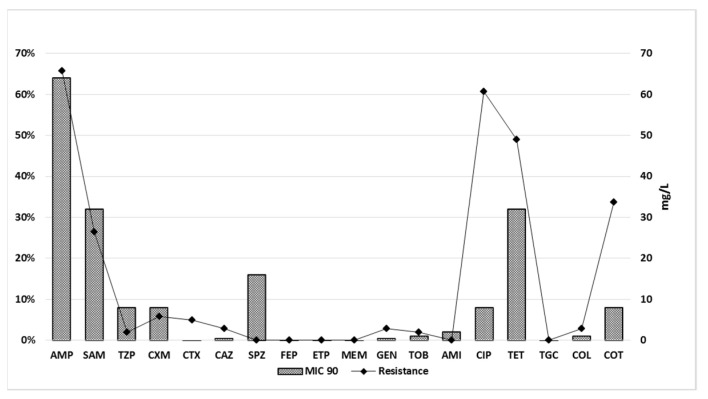
The values of percentage of resistance and MIC 90 in *E. coli* of asymptomatic broilers. Abbreviations: AMP = ampicillin; SAM = ampicillin + sulbactam; TZP = piperacillin + tazobactam; CXM = cefuroxime; CTX = cefotaxime; CAZ = ceftazidime; SPZ = cefoperazone + sulbactam; FEP = cefepime; ETP = ertapenem; MEM = meropenem; GEN = gentamicin; TOB = tobramycin; AMI = amikacin; CIP = ciprofloxacin; TET = tetracycline; TGC = tigecycline; COL = colistin and COT = trimethoprim + sulphonamide.

Antimicrobial testing showed that the most prevalent resistance in broilers was to ampicillin (66%; MIC 90 (minimum inhibitory concentration required to inhibit the growth of 90% of microorganisms) = 64 mg/L), followed by ciprofloxacin (61%; MIC 90 = 8 mg/L), tetracycline (49%; MIC 90 = 32 mg/L), trimethoprim + sulphonamide (34%; MIC 90 = 8 mg/L), and ampicillin + sulbactam (27%; MIC 90 = 32 mg/L). In eleven isolates, resistance to cephalosporins was detected, namely, to cefuroxime (5.88%; MIC 90 = 8 mg/L) and cefotaxime (4.9%; MIC 90 = 0.25 mg/L). Reduced sensitivity to tazobactam, ceftazidime, gentamicin, tobramycin, and colistin was found in less than 3% of bacterial isolates (Figure 1).

Multi-drug resistance (MDR), i.e., resistance to three and more antibiotics from different groups, occurred in seven strains from broilers reared on commercial farms, which was 6.1%. The MDR strains were identified in phylogroups A, B1, B2, and D. Of all tested strains, 76 were antibiotic-resistant. In general commensal groups, A, B1, and C comprised more than half of the resistant strains, specifically 53.9% altogether (A = 17.1%, B1 = 35.5%, C = 1.3%), in comparison to pathogenic groups (39.5%). Of resistant strains, 6.6% were not classified in any phylogroup (Table 2).

Interpretative reading revealed the following mechanisms of resistance: TEM-1,2/SHV-1:low (approx. 18%); TEM-1,2/SHV-1:high (42%). Other mechanisms detected in broilers were extended-spectrum β-lactamases (ESBL) in 6%, of which ESBL TEM was in less than 4% and ESBL CTX-M type in <2%. The other important mechanisms were AGL AAC(6′)I, i.e., incomplete fluoroquinolone cross-resistance conferring resistance also to kanamycin (1.96%), and AGL ANT(2′)I (0.98%), i.e., cross-resistance between some aminoglycosides. Based on the above results, we focused on monitoring the gene conferring resistance to cephalosporins (*bla_CTX-M_*); fluoroquinolones (*qnrA, qnrB, qnrS, aac(6′)-Ib-cr*); tetracyclines (*tetA, tetB*); sulfonamides (*sul1, sul2);* aminoglycosides (*aadA*); trimethoprim *(dfrA, dfrB*); and plasmid-mediated colistin resistance (*mcr 1–2*). The most interesting resistant and virulent patterns are shown in Table 3. Considering the results, we should also mention the presence of genes for resistance to cephalosporins (*bla_CTX-M-1_, bla_CTX-M-2_*). A positive result was the non-presence of resistance to carbapenems (ertapenem, meropenem).

## 3. Discussion

In our study, we found six virulence-associated genes of ExPEC in isolates from the intestinal microbiota of broilers, namely, *tsh* (9.6%), *papC* (4.3%), *cvaC* (21.7%), *iss* (29.6%), *kpsMTII* (30.4%), and *ibeA* (3.5%). The percentages indicate that the most numerous virulence factors were protectins. These protectins are typical for avian pathogenic *E. coli* (APEC), and they also contribute to survival and proliferation of microorganisms in the host [9]. Wang et al. [10] analysed the presence of invasion by brain endothelium protein A in isolates from ducks with colibacillosis, but none were found in healthy birds. Our results showed the presence of *ibeA* gene only in 3.5% of strains from healthy (asymptomatic) chickens, belonging to groups B2 and D. The recent study by Meena et al. [11] testifies to the presence of the genes characteristic for ExPEC (*iroN, cvaC,* and *kpsMTII*) more often in groups B2 and D, while *papC* gene was distributed among all phylogroups. Our results show *iroN* mainly in B1; *kpsMTII* in D; and *cvaC* and *papC* equally in broiler B2 and D groups. The strains containing genes for the synthesis of capsule and P fimbrie were classified in the ExPEC pathotype. The production of adhesins (fimbriae/pilli) is one of the virulence factors in UPEC enabling the colonization, adherence, and creation of the host inflammatory response [12]. P fimbriae coded by the *papC* gene, localized at the cell surface, are associated with upper urinary tract infections by binding to the endothelium of the kidney vascular system. Colonizing of the urinary tract by UPEC associated with *papC* gene can lead to pyelonephritis [13]. We found the *papC* gene in a total of five strains from broilers, of which three were UPEC (group B2), one was APEC (D group), and the remaining strain was ExPEC (unknown group).

*E. coli* strains colonizing human and animal guts can acquire genes coding virulence factors from pathogenic strains and can cause intestinal or extra-intestinal infections in various organ systems (specifically, urinary tract infections, pneumonia, meningitis, and sepsis). Köhler and Dobrindt [14] characterized virulence factors typical for extra-intestinal pathogenic *E. coli* (ExPEC), such as various adhesins (e.g., S/F1C and P fimbriae), toxins (e.g., cytotoxic necrotizing factor 1), factors for eliminating the host defense system (e.g., increased serum survival, colicin V, and capsule synthesis), and for nutrition acquisition (e.g., siderophores and their receptors). These virulence factors are often carried on mobile genetic elements such as pathogenity islands (PAI), which help to transport genes between commensals and pathogens. Iron availability modulates the gut microbiota composition [15]. Bacterial iron recovery is thus also one of the virulence factors available to bacteria. One method of getting iron from the environment is through the production of specific iron chelators, called siderophores, and their receptors on cell surfaces [16]. Tu et al. [17] investigated the influence of iron-uptake genes *fyuA* and *irp2* on APEC pathogenesis. Deletion of these genes from the genome led to reduced transcription of virulence genes and their ability to adhere to cells. In general, pathogenicity was more reduced through the absence of *fyuA* than in strains with the deletion of *irp2*. These authors summarized the hypothesis about the cooperation of *irp2* and *fyuA* in APEC pathogenicity, which can be the reason for the presence of *irp2* gene in all isolates containing gene *fyuA* analysed in the present study. The iron acquisition systems used in low-iron conditions are encoded by *sitA, iroN*, and *iutA* genes. More than half of the strains from broilers and goshawks possessed these genes in the research by Handrová and Kmeť [18]. Our results point to the presence of all three genes in *E. coli* strains isolated from broilers. The Feo system is able to transport Fe^2+^, which occurs in anaerobic conditions and at low pH and which is necessary for bacteria living in such environments. Proteins FeoB and FeoA, which are components of the Feo system, participated in bacterial virulence [19]. The *feoB* together with *sitA* were the most common genes identified by us (on average, around 90%) in broilers.

Previous studies have demonstrated the function of phylogroups. Groups A and B1 form the commensal microbiota and B2 and D are connected with extra-intestinal infections, and they are also called pathogenic groups [20]. The strains isolated from healthy chickens were classified mainly as commensal groups B1 (36.5%) and A (20.9%). APEC with UPEC are part of the *E. coli* group responsible for extra-intestinal infections. The strain classified as ExPEC must present two or more virulence factors including P fimbriae (*papA, papC*), S/F1C fimbriae (*sfa/foc*), Dr-binding adhesins (*afa/dra*), capsule synthesis (*kpsMT*), and aerobactin receptor (*iutA*) [6]. Of all our isolated *E. coli* strains (*n* = 115), 40 were included in pathotype ExPEC and the remaining 75 in non-ExPEC. The distribution of phylogroups among ExPEC and non-ExPEC was different. The ExPEC pathotype includes strains belonging in groups B2 (15%), D (27.5%), F (17.5%), clade I (2.5%), A (22.5%), and B1 (10%). However, in non-ExPEC, the dominant groups were B1 (50.7%) and A (20%) versus D (12%), clade I (5.3%), C (2.7%), and B2 (1.3%). Virulence factors typical for ExPEC were more often detected in groups B2 and D.

A large diversity of antimicrobials is used to raise poultry in most countries, and most of them are considered to be essential in human medicine [21]. The study conducted by Joosten et al. [22] reported that aminopenicillins (ampicillin and amoxicilin), fluoroquinolones, and tetracycline were the most frequently used antimicrobials in broilers in nine European countries, and only 3 to 26% of drugs were of the “highest priority critically important for human health” group of drugs (including colistin, quinolones, cephalosporins, and macrolides). This roughly reflects the situation in our farms, where the occurrence of resistance was as follows: the highest resistance was to ampicillin followed by ciprofloxacin, tetracycline, and ampicillin + sulbactam. The resistance to cephalosporins, aminoglycosides, and polymyxins was detected at less than 6% of isolates.

In our study, we detected altogether 76 strains (66.1%) resistant to at least one antibiotic. Multidrug-resistant strains represented 6.1% (seven strains).

Johar et al. [23] analysed resistance of the APEC strain in healthy and non-healthy chickens. The resistance to ampicillin, cephalothin, ciprofloxacin, tetracycline, and fosfomycin was higher than 75% in both bird groups. Isolates from sick birds showed resistance to cefuroxime, ceftriaxone (4.4%), piperacillin-tazobactam (1.5%), and colistin (33.3%). Resistance to cephalosporins, namely, cefuroxime, cefotaxime, and ceftazidime, was found in 5.88%, 4.9%, and 2.94% of birds, respectively and to piperacillin-tazobactam (1.96%) in healthy broilers in our study. Of broilers, 2.94% demonstrated phenotypic resistance to colistin, but the value of MIC 90 was 1.0 mg/L, i.e., less than what is listed in EUCAST clinical breakpoints (2.0 mg/L). The resistance of *E. coli* from broilers in EU member states was analysed and evaluated by the European Food Safety Authority (EFSA) and the European Centre for Disease Prevention and Control (ECDC). Their results showed that the average of resistance to cephalosporins was 3%, with 8.5% in Spain, 3% in France and Poland, and 1% in Germany [24]. In another study from Italy, the susceptibility of *E. coli* from various poultry sources was investigated. The results confirmed the resistance to cephalosporins of the first (cefazolin—16%), second (cefoxitin—2%), and third (ceftazidime—2%) generation [25]. The results of these studies are consistent with our findings regarding cephalosporins in broilers (cefuroxime—5.88%, cefotaxime—4.9%, and ceftazidime—2.94%). Higher percentages detected by us represent resistance to ampicillin (65.69% of broilers), ampicillin-sulbactam (26.47%), tetracycline (49.02%), and trimethoprim-sulphonamide (33.66%). Tetracycline is commonly used in poultry treatment, and this is the main reason for the high resistance to this antibiotic [26]. *E. coli* isolates from southwestern Nigeria were resistant to tetracycline in 81% of samples, which was the highest level found in this study [27]. In our findings, we detected resistance to tetracyclines as the third highest resistance in broilers (49.02%). *E. coli* isolated from broilers reared on farms in the Netherlands were resistant to ciprofloxacin in 50% of cases, in comparison to turkeys (45%) and laying hens (0%) [28]. Reduced sensitivity to ciprofloxacin was confirmed in our study too, namely, in 60.78% of broilers. Resistance to aminoglycosides was confirmed in commercial poultry in Nepal, where seven strains isolated from laying hens were resistant to gentamicin. On the other hand, broilers had not developed this resistance. Resistance to amikacin was not detected in any of the samples [29]. The *E. coli* from broilers in our study showed resistance to gentamicin in 2.94% and to tobramycin in 1.96% of samples. Two chicken isolates (1.96%) were detected as susceptible with increased exposure to amikacin.

Genotypic resistance was analysed only in some strains according to the results of our interpretive reading.

Cefotaximases confer resistance against all β-lactam antibiotics apart from cephamycins and carbapenems. Furthermore, CTX-M-producing *E. coli* strains are often resistant to other families of antibiotics such as quinolones, aminoglycosides, or cotrimoxazole. This remains a major emerging health concern because the choice of effective antimicrobial drugs is limited [30]. We found similar results as follows: CTX-M-producing *E. coli* were confirmed in two broilers. One of them was positive for presence of the gene *bla_CTX-M-1_* and the second for *bla_CTX-M-2_*. One strain had a gene encoding resistance to aminoglycosides; both of them were phenotypically resistant to ciprofloxacin and trimetoprim-sulfonamide. The *Enterobacterales* family often has a connection between plasmid-mediated quinolone resistance and extended-spectrum β-lactamases (ESBL) mechanisms [31]. The presence of *aac(6′)-Ib-cr* was confirmed in both strains from broilers with an identified mechanism of resistance. The acquisition of resistance leads to the formation of multi-drug-resistant strains and to the limitation of the effects of antimicrobial therapy [32].

Reduced sensitivity to carbapenems was not confirmed in any of our isolates. This is positive information for human medicine, because of their use as a last resort in treating bacterial infections, together with polymyxin colistin as the last choice in the treatment of human infections caused by carbapenem-resistant enterobacteria. The development of resistance to these antibiotics is therefore a global threat to the treatment of bacterial infections in humans and animals [33]. Cepas and Soto [34] described the relationship between growing resistance and a reduced amount of virulence factors. One possible hypothesis is that the process of acquisition of antimicrobial resistance is connected with deletion of virulence-associated genes from DNA regions. Several studies have focused on investigating UPEC strains and their susceptibility to quinolones and virulence. The strains resistant to quinolones did not possess virulence factors such as aerobactin and P-fimbriae [35]. Furthermore, *E. coli* strains producing haemolysins were more susceptible to tetracycline, nalidixic acid, cefotaxime, and cotrimoxazol than isolates without haemolysin production [36]. High resistance and low virulence were observed in non-B2 strains, and, in contrast, the B2 group had wide virulence factor capacity in isolates in a study carried out in French hospitals [37]. Our *E. coli* isolates of broiler origin presented higher average numbers of virulence factors, specifically 7.9, 7.6, and 4.1 in groups F, B2, and D, respectively. Less virulent were strains from groups A and B1, which accounted for approximately 3.0 factors. Despite the fact that most of the virulence genes were found in the pathogenic phylogroups, their susceptibility was higher compared to the commensal ones. In contrast, more than half (52.6%) of the resistant strains were groups A and B1, but they were poor in terms of virulence. This can be a reason for these strains’ survival and growth in an antimicrobial environment, whereas their ability to survive in normal conditions can be limited.

## 4. Materials and Methods

### 4.1. Samples, Bacterial Isolation, and Identification

A total of 115 *E. coli* strains isolated from cloacal swabs of asymptomatic broilers from commercial farms were analysed in this study. The samples were collected from five broiler commercial farms, which are situated in regions of the North and the West Slovakia. All chickens were 12–30 days of age and reared in a large-capacity farm with litter floor rearing. In our study were examined the broiler breed Ross 308. Samples were inoculated in buffered peptone water (Oxoid, Basingstoke, UK) at 37 °C for 12 h and then cultured on MacConkey (Oxoid, Basingstoke, UK) and Uriselect agar (Bio-Rad Laboratories, Hercules, CA, USA) under the same temperature conditions overnight. Individual colonies were identified as *E. coli* using a matrix-assisted laser desorption/ionization time-of-flight mass spectrophotometry (MALDI-TOF MS) biotyper (Bruker Daltonics, Leipzig, Germany), according to the method described by Bessède et al. [38].

### 4.2. DNA Extraction, Clermont’s Phylogenetic Typing, and Detection of Iron Uptake and Virulence-Associated Genes

Genomic DNA used for polymerase chain reaction (PCR) analysis was extracted from overnight culture by means of the boiling DNA extraction method. Briefly, samples were centrifuged at 12,000× *g* for 15 min, the supernatant was eliminated, and then the pellets were washed with filtered distilled water. Then, the pellets were re-suspended in 200 µL of filtered distilled water, subjected to boiling at 100 °C in a heat block for 10 min, cooled on ice for 10 min, and centrifuged for 2 min at 12,000× *g*, and then the supernatant was used for PCR. The isolated DNA was quantitatively and qualitatively analysed with a Nanodrop 2000c Spectrofotometer (Thermo Fisher Scientific, Wilmington, DE, USA) and stored at −20 °C.

The isolates were subjected to phylogenetic typing using the quadruplex phylogroup assignment method [39] for detection of eight *E. coli* phylogroups. The protocols are based on amplification of *chuA*, *yjaA*, *arpA*, and *TspE4.C2* DNA fragments and additional testing for specific genes in the E (*arpAgpE*) and C (*trpAgpC*) groups. All detected genes, their primer sequences, PCR products sizes, annealing temperatures, and relevant references used in PCR are listed in Table 4.

Multiplex and/or single PCR analysis was also used to confirm the presence of iron acquisition genes including *iha*-hemaglutinin adhesine; *iroN*-siderophore salmochelin; *fyuA*-siderophore yersiniabactin; *ireA*-iron-responsive element; *sitA*-iron and manganese transport; *feoB*-Fe^2+^ transporter; *irp2*-iron regulatory protein and virulence genes: *iss*-increased serum survival gene; *papC*-fimbrial adhesine; *iutA*-siderophore aerobactin; *cvaC*-colicin V; *kpsMTII*-capsule; and *tsh*-temperature-sensitive hemaglutinin; *ibeA*-invasion of brain (Table 4).

The reaction mixture for PCR in each tube contained: 2 μL DNA; 0.2 μL each of the 0.1–1 μM primers; 0.2 μL 1U Taq DNA polymerase; 1.5–2 μL 1.5 mM MgCl_2_; 2.5 μL 200 μM dNTP; and 2.5 μL 10x reaction buffer in a total volume of 25 μL. The conditions for running the PCR reaction were: initial denaturation (95 °C, 10 min), 30 cycles of denaturation (95 °C, 20–30 s), annealing (50–65 °C, depending on the primers, 30–60 s), extension (72 °C, 30–60 s), and final elongation (72 °C, 5–10 min).

PCR amplicons were visualized on 1.5% agarose gel prepared in 1× TAE buffer coloured with GoodViewTM (Beijing SBSGenetech Co., Ltd., Beijing, China) using a Mid-Range DNA Ladder (Jena Bioscience, Jena, Germany). The DNA fragments were visualized with an ultraviolet lamp (UV Transilluminator, Geno View, VWR, Radnor, PA, USA).

### 4.3. Antimicrobial Susceptibility Testing

Antibiotic susceptibility was determined by means of minimum inhibitory concentration (MIC) testing performed according to Gattringer et al. [60] using the Bel-MIDITECH automated diagnostic system (Bratislava, Slovakia). This diagnostic system consists of antimicrobial drugs: ampicillin (AMP); ampicillin + sulbactam (SAM); piperacillin + tazobactam (TZP); cefuroxime (CXM); cefotaxime (CTX); ceftazidime (CAZ); cefoperazone + sulbactam (SPZ); cefepime (FEP); ertapenem (ETP); meropenem (MEM); gentamicin (GEN); tobramycin (TOB); amikacin (AMI); tigecycline (TGC); ciprofloxacin (CIP); tetracycline (TET); colistin (COL); and trimethoprim + sulfonamide (COT). The results of MIC values for each antibiotic were interpreted according to clinical breakpoints (CBPs) described in EUCAST 2020 [61].

### 4.4. Detection of Antimicrobial Resistance Genes

The detection of various antimicrobial resistance genes was carried out with a PCR assay. We detected genes encoding plasmid-mediated quinolone resistance (PMQR): *qnrA, qnrB, qnrS*, and *aac(6′)IbCr* and genes conferring resistance to polymyxin (*mcr1, mcr2*); cephalosporins (*bla_CTX-M_*); tetracycline (*tetA, tetB*); sulfonamide (*sul1* and *sul2*); aminoglycosides (*aadA*); trimethoprim (*dfrA, dfrB*); *integrase 1* (*Int1*); and transposon (*Tn3*) (Table 4).

## 5. Conclusions

In conclusion, our results indicate that broilers reared for human consumption can be considered as potential reservoirs and carriers of ExPEC strains, containing high numbers of virulence-associated genes with the possibility of causing infections in both humans and animals. Some of these isolates were resistant to antimicrobials used in human treatments, such as cephalosporines and fluoroquinolones (presence of *E. coli* with CTX-M; and plasmid quinolone resistance *qnrS; aac(6′)-Ib-cr*), and 6.1% of broiler-associated isolates showed multi-drug resistance. In addition, the high prevalence of mobile elements (*Int1* and *Tn3*) may allow gene dissemination. Based on these findings, we conclude that commercial broilers can be a potential threat for other animals and humans, because of the potential for meat and other products to become contaminated due to non-compliance with hygiene practices in farming or food-handling.

## Figures and Tables

**Table 1:** Distribution of individual pathotypes related to phylogenetic groups in broilers.

Pathotype	Number (%) of All Samples	A	B1	B2	D	F	Clade I	Unknown
ExPEC	25 (21.7)	9 (36.0)	2 (8.0)	0 (0)	9 (36.0)	4 (16.0)	0 (0)	1 (4.0)
UPEC	8 (7.0)	0 (0)	0 (0)	6 (75.0)	2 (25.0)	0 (0)	0 (0)	0 (0)
APEC	7 (6.1)	0 (0)	2 (28.6)	0 (0)	0 (0)	3 (42.9)	1 (14.3)	1 (14.3)

Abbreviations: ExPEC = extra-intestinal pathogenic *E. coli*; UPEC = uropathogenic *E. coli*; APEC = avian pathogenic *E. coli*.

**Table 2:** Antibiotic resistant isolates of commercial broilers associated with phylogroups.

Phylogroup	Resistant Strains	
	*n*	*%*
A	13	17.1
B1	27	35.5
B2	5	6.6
C	1	1.3
D	16	21.1
F	7	9.2
Clade I	2	2.6
Unknown	5	6.6
Total	76	100

Abbreviations: *n* = number of isolates.

**Table 3:** Combination of virulence factors and phenotypic and genotypic resistance in selected strains.

	Strain Group	Mobilome	Virulence	Phenotypic Resistance	Genotypic Resistance
Commensals	B-1 B1	*Int1, Tn3*	*feoB*	AMP, SAM, CXM, CAZ, TOB, CIP, COT	*aac(6′)-Ib-cr, sul1, dfrA*
	B-2 A	*Int1, Tn3*	*feoB*	AMP, SAM, CXM, CTX, CAZ, CIP, COT	*aac(6′)-Ib-cr, sul1, dfrA*
	B-13 B1	*Tn3*	*feoB, sitA, iroN, iss*	AMP, CXM, CTX, CIP, TET, COT	*tetA,* *bla_CTX-M-2_*
	B-23 A		*feoB, sitA, iutA, iroN, tsh, iss, cvaC*	AMP, SAM, CIP	*qnrS*
UPEC	B-22 B2	*Int1, Tn3*	*sitA, chuA, iutA, irp2, fyuA, papC, cvaC*	AMP, CXM, CTX, CIP, TET, COT	*aadA, sul2, tetB, dfrA,* *bla_CTX-M-1_*
	B-42 B2		*feoB, sitA, chuA, iutA, iroN, ibeA, irp2, fyuA, tsh, iss, cvaC*	Sensitive	
	B-21 B2	*Tn3*	*feoB, sitA, chuA, iutA, irp2, fyuA, papC, cvaC*	AMP, CIP, TET	*aadA, sul2, tetB, dfrA*
	B-32 D	*Int1, Tn3*	*feoB, sitA, chuA, iutA, irp2, fyuA, kpsMTII*	AMP, SAM, CIP, COT	*aadA*
APEC	B-31 F	*Tn3*	*feoB, sitA, chuA, iutA, iroN, irp2, tsh, iss, cvaC, kpsMTII*	AMP, SAM, CIP, TET	*qnrS, tetA*
	B-71 clade I	*Int1, Tn3*	*feoB, sitA, chuA, iutA, iroN, irp2, fyuA, papC, cvaC, kpsMTII*	AMP, CIP, COT	*aadA, sul2, tetB*
ExPEC	B-93 D	*Int1, Tn3*	*feoB, sitA, chuA, iutA, ibeA, iss, kpsMTII*	AMP, GEN, TOB, CIP, TET, COT	*aadA, sul1, sul2, tetA, dfrA*

Abbreviations: UPEC = uropathogenic *E. coli*; APEC = avian pathogenic *E. coli*; ExPEC = extra-intestinal pathogenic *E. coli*; B = broiler; genes encode mobilome, virulence, and resistance, and abbreviations of antibiotics are mentioned in the “Materials and Methods” section.

**Table 4:** Target genes and primers sequences used in the PCRs performed in this study to determine the *E. coli* phylogroups, iron uptake system, virulence factors, and antimicrobial resistance.

Gene	Primer Sequences (5′–3′)	Product (bp)	Annealing (°C)	References
*arpA*	AACGCTATTCGCCAGCTTGC TCTCCCCATACCGTACGCTA	400	59	[39]
*chuA*	ATGGTACCGGACGAACCAAC TGCCGCCAGTACCAAAGACA	288	59	[39]
*yjaA*	CAAACGTGAAGTGTCAGGAG AATGCGTTCCTCAACCTGTG	211	59	[39]
*TspE4.C2*	CACTATTCGTAAGGTCATCC AGTTTATCGCTGCGGGTCGC	152	59	[39]
*arpA* (group C)	GATTCCATCTTGTCAAAATATGCC GAAAAGAAAAAGAATTCCCAAGAG	301	57	[40]
*trpA* (group E)	AGTTTTATGCCCAGTGCGAG TCTGCGCCGGTCACGCCC	319	59	[40]
*iha*	CTGGCGGAGGCTCTGAGATCA TCCTTAAGCTCCCGCGGCTGA	827	60	[41]
*iroN*	AAGTCAAAGCAGGGGTTGCCCG GACGCCGACATTAAGACGCAG	655	60	[41]
*fyuA*	TGATTAACCCCGCGACGGGAA CGCAGTAGGCACGATGTTGTA	880	55	[42]
*ireA*	TGGTCTTCAGCTATATGG ATCTATGATTGTGTTGGT	415	55	[43]
*sitA*	AGGGGGCACAACTGATTCTCG TACCGGGCCGTTTTCTGTGC	608	59	[44]
*feoB*	AATTGGCGTGCATGAAGATAACTG AGCTGGCGACCTGATAGAACAATG	470	59	[45]
*irp2*	AAGGATTCGCGTGAC TCGTCGGGCAGCGTTTCTTCT	287	59	[45]
*iss*	ATCACATAGGATTCTGCCG ACAAAAAGTTCTATCGCTTCC	700	61	[46]
*papC*	GACGGCTGTACTGCAGGGTGTGGCG ATATCCTTTCTGCAGGGATGCAATA	328	61	[47]
*iutA*	GGCTGGACATGGGAACTGG CGTCGGGAACGGGTAGAATCG	300	63	[42]
*cvaC*	CACACACAAACGGGAGCTGTT CACACACAAACGGGAGCTGTT	680	63	[42]
*tsh*	GGTGGTGCACTGGAGTGG AGTCCAGCGTGATAGTGG	620	55	[48]
*ibeA*	TGAACGTTTCGGTTGTTTTG TGTTCAAATCCTGGCTGGAA	814	55	[49]
*kps MTII*	GCGCATTTGCTGATACTGTTG CATCCAGACGATAAGCATGAGCA	272	63	[42]
*qnrA*	ATTTCTCACGCCAGGATTTG GATCGGCAAAGGTTAGGTCA	516	53	[50]
*qnrB*	GATCGTGAAAGCCAGAAAGG ACGATGCCTGGTAGTTGTCC	469	53	[50]
*qnrS*	ACGACATTCGTCAACTGCAA TAAATTGGCACCCTGTAGGC	417	53	[50]
*aac(6)-Ib-cr*	GATCTCATATCGTCGAGTGGTGG GAACCATGTACACGGCTGGAC	435	58	[50]
*mcr-1*	CGGTCAGTCCGTTTGTTC CTTGGTCGGTCTGTAGGG	309	58	[51]
*mcr-2*	TGTTGCTTGTGCCGATTGGA AGATGGTATTGTTGGTTGCTG	567	58	[52]
*bla_CTX-M-1_*	AAAAATCACTGCGCCAGTTC AGCTTATTCATCGCCACGTT	415	52	[53]
*bla_CTX-M-2_*	CGACGCTACCCCTGCTATT CCAGCGTCAGATTTTTCAGG	552	52	[53]
*tetA*	GGCCTCAATTTCCTGACG AAGCAGGATGTAGCCTGTGC	372	55	[54]
*tetB*	GAGACGCAATCGAATTCGG TTTAGTGGCTATTCTTCCTGCC	228	55	[54]
*sul1*	CGGCGTGGGCTACCTGAACG GCCGATCGCGTGAAGTTCCG	433	69	[55]
*sul2*	GCGCTCAAGGCAGATGGCATT GCGTTTGATACCGGCACCCGT	293	69	[55]
*aadA*	TGATTTGCTGGTTACGGTGAC CGCTATGTTCTCTTGCTTTTG	284	60	[56]
*dfrA*	GTGAAACTATCACTAATGG TTAACCCTTTTGCCAGATTT	474	55	[57]
*drfB*	GATCGCCTGCGCAAGAAATC AAGCGCAGCCACAGGATAAAT	141	60	[57]
*Int1*	GGGTCAAGGATCTGGATTTCG ACATGCGTGTAAATCATCGTCG	483	62	[58]
*Tn3*	CACGAATGAGGGCCGACAGGA ACCCACTCGTGCACCCAACTG	500	58	[59]

Abbreviations: *arpA, chuA, yjaA, TspE4.C2, arpA gp.C, trpA gp.E* = identification of phylogroups; *iha* = hemaglutinin adhesine; *iroN* = siderophore salmochelin; *fyuA* = siderophore yersiniabactin; *ireA* = iron-responsive element; *sitA* = iron transport; *feoB* = Fe^2+^ transporter; *irp2* = iron regulatory protein; *iss* = increased serum survival gene; *papC* = fimbrial adhesine; *iutA* = siderophore aerobactin; *cvaC* = colicin V; *tsh* = temperature-sensitive hemagglutinin; *ibeA* = invasion of brain endothelium; *kps MTII* = capsule synthesis; *qnrA, qnrB, qnrS, aac(6)-Ib-cr,* = quinolone resistance; *mcr-1, mcr-2* = resistance to colistin; *bla_CTX-M-1_*, *bla_CTX-M-2_* = cephotaximases; *tetA, tetB* = resistance to tetracycline; *sul1, sul2* = resistance to sulfonamide; *aadA* = aminoglycoside resistance; *dfrA, dfrB* = resistance to trimepthoprim; *Int1* = integrase 1; *Tn3* = transposon.

## Data Availability

Not applicable.
